# Implementation of Machine Learning Applications in Health Care Organizations: Systematic Review of Empirical Studies

**DOI:** 10.2196/55897

**Published:** 2024-11-25

**Authors:** Luigi M Preti, Vittoria Ardito, Amelia Compagni, Francesco Petracca, Giulia Cappellaro

**Affiliations:** 1 Center for Research on Health and Social Care Management (CERGAS) SDA Bocconi School of Management Milan Italy; 2 Department of Social and Political Sciences Bocconi University Milan Italy

**Keywords:** artificial intelligence, machine learning, implementation, health care organization, barriers, facilitators

## Abstract

**Background:**

There is a growing enthusiasm for machine learning (ML) among academics and health care practitioners. Despite the transformative potential of ML-based applications for patient care, their uptake and implementation in health care organizations are sporadic. Numerous challenges currently impede or delay the widespread implementation of ML in clinical practice, and limited knowledge is available regarding how these challenges have been addressed.

**Objective:**

This work aimed to (1) examine the characteristics of ML-based applications and the implementation process in clinical practice, using the Consolidated Framework for Implementation Research (CFIR) for theoretical guidance and (2) synthesize the strategies adopted by health care organizations to foster successful implementation of ML.

**Methods:**

A systematic literature review was conducted based on the PRISMA (Preferred Reporting Items for Systematic Reviews and Meta-Analyses) guidelines. The search was conducted in PubMed, Scopus, and Web of Science over a 10-year period (2013-2023). The search strategy was built around 4 blocks of keywords (artificial intelligence, implementation, health care, and study type). Only empirical studies documenting the implementation of ML applications in clinical settings were considered. The implementation process was investigated using a thematic analysis and coding procedure.

**Results:**

Thirty-four studies were selected for data synthesis. Selected papers were relatively recent, with only 9% (3/34) of records published before 2019. ML-based applications were implemented mostly within hospitals (29/34, 85%). In terms of clinical workflow, ML-based applications supported mostly prognosis (20/34, 59%) and diagnosis (10/34, 29%). The implementation efforts were analyzed using CFIR domains. As for the inner setting domain, access to knowledge and information (12/34, 35%), information technology infrastructure (11/34, 32%), and organizational culture (9/34, 26%) were among the most observed dimensions influencing the success of implementation. As for the ML innovation itself, factors deemed relevant were its design (15/34, 44%), the relative advantage with respect to existing clinical practice (14/34, 41%), and perceived complexity (14/34, 41%). As for the other domains (ie, processes, roles, and outer setting), stakeholder engagement (12/34, 35%), reflecting and evaluating practices (11/34, 32%), and the presence of implementation leaders (9/34, 26%) were the main factors identified as important.

**Conclusions:**

This review sheds some light on the factors that are relevant and that should be accounted for in the implementation process of ML-based applications in health care. While the relevance of ML-specific dimensions, like trust, emerges clearly across several implementation domains, the evidence from this review highlighted that relevant implementation factors are not necessarily specific for ML but rather transversal for digital health technologies. More research is needed to further clarify the factors that are relevant to implementing ML-based applications at the organizational level and to support their uptake within health care organizations.

**Trial Registration:**

PROSPERO 403873; https://www.crd.york.ac.uk/prospero/display_record.php?RecordID=403873

**International Registered Report Identifier (IRRID):**

RR2-10.2196/47971

## Introduction

### Background

Artificial intelligence (AI) has been unquestionably acknowledged as a game changer in health care [1], even more so after technological advances in the field of machine learning (ML) have contributed to further expand the frontiers of its possible applications [2]. Compared to knowledge- or rule-based systems that automate established human clinical reasoning methods through a series of “if-then” statements [3], ML encompasses all nonknowledge-based models that automatically (or semiautomatically) learn from the exposure to abundant quantities of data and detect patterns through explicit or latent recognition rather than conventional programming. ML is expected to serve primarily as a decision support tool to enhance human work rather than replace it [4], thereby providing health care professionals (HCPs) with improved predictions and rendering their decision-making process more accurate [5]. Although some AI systems have already been shown to be equal or even superior in performance to HCPs [6], full automation of a broad range of human tasks is expected to occur only at later stages.

Irrespective of whether ML is intended to provide inputs to human decision-making or to act autonomously, these technological advancements do not automatically translate into clinical practice. The road to implementing ML applications in patient care has several challenges, creating an inevitable chasm between ML and its clinical integration [7,8].

Challenges for the implementation of AI systems, without an exclusive focus on ML, have been previously outlined, with various interdependent factors at different stakeholder group levels [9,10]. For HCPs, core considerations pertain to the need for ML outputs to be meaningful inputs in their decision-making and be explainable. ML algorithms are often associated with the so-called “black box” effect [11,12]. The lack of transparency in data and outputs can be a significant concern for HCPs, as it hampers model interpretability (ie, the possibility to understand or interpret how a given output has been produced) and explainability (ie, the capacity of a model to be explained, even if not totally interpretable) [13]. ML applications and outputs are therefore likely to clash with the principles of evidence-based medicine, which instead involve the highest possible standards of interpretability and explainability. Concerns about the potential implications for accountability and personal responsibility regarding mistakes or computational misdiagnosis by ML applications present additional implementation challenges.

At the patient level, fair implementation of ML applications necessitates continuous supply of standardized data to train, validate, and incessantly improve performance and prevent algorithmic bias [9]. Notions of patient confidentiality and privacy should be reimagined entirely as data must be shared across multiple institutions to maximize their value and allow for improved algorithms [14].

Lastly, distinctive implementation challenges have been identified at the level of health care organizations, which are associated with financial challenges and funding mechanisms, as well as issues related to the computational resources that are necessary to support the implementation of ML.

Several implementation frameworks for health care technologies are on hand, but no widely recognized model addresses all the specific issues that are relevant to ML applications [15-17]. To date, research on ML implementation has been predominantly conceptual in nature, with an underreporting of empirical investigations into the specifics and consequences of implementation processes in real-life settings [18,19]. Available studies have primarily focused on the quantitative impact of ML algorithms on health outcomes or accuracy, without examining the corresponding implementation processes [20]. Recently, Chomutare et al [21] conducted a scoping review to identify barriers and facilitators to the implementation of ML from empirical studies, while Tricco et al [22] focused on the strategies adopted to implement ML tools in hospital settings. However, additional inquiry is needed to determine whether the literature on the implementation of ML applications in health care adequately acknowledges the unique challenges encountered along the implementation process, as well as the strategies adopted to overcome them.

### Research Objectives

This systematic literature review primarily aims to identify studies on the real-life implementation of ML applications in clinical practice and to synthesize insights about the features of these innovations and the processes deployed to facilitate their effective implementation. We set out to address the following research questions:

1. What are the characteristics of ML applications implemented in clinical practice as reported in the scientific literature?
2. What processes and strategies do health care organizations employ to foster the successful implementation of ML applications in clinical practice? Which factors are recognized as more relevant for the unsuccessful implementation of ML applications?

## Methods

### Overview

This systematic review adopted the PRISMA (Preferred Reporting Items for Systematic Reviews and Meta-Analyses) 2020 guidelines (Multimedia Appendix 1) [23]. The review was previously registered within the International Prospective Register of Systematic Reviews (PROSPERO) with registration number 403873. All methodological details are provided in the published research protocol [24]. The most relevant aspects are summarized hereafter, with any deviations from the protocol duly noted.

### Positionality of the Research Team

Positionality refers to how individuals identify with and relate to different social dimensions such as gender, race, and ethnicity [25], and as such is a relevant aspect to consider in qualitative research. To that end, the research team comprised 5 Italian white researchers (LMP, VA, AC, FP, and GC). Broadly speaking, the team as a whole included 60% females and 40% males and shared a common background in management studies with a focus on health care management. LMP is a PhD student who is working in the areas of AI and ML under the perspective of the organizational implementation of AI tools in health care organizations. VA is a PhD student who has conducted prior research at the intersection between digital health and implementation science. AC has multi-annual experience in organizational studies and qualitative research focusing on issues related to innovations in health care and professional dynamics in health care organizations. FP is a PhD student who is an expert in digital health technologies, focusing on their regulation and value assessment. GC has multi-annual experience in organizational studies and qualitative research focusing on institutional dynamics, novel technologies, and professions.

### Eligibility Criteria

This review focused on empirical studies investigating aspects related to the implementation of ML applications within health care organizations. We adopted the definition of implementation as an “active and planned effort to mainstream innovation within an organization” [26], while health care organizations encompassed all entities delivering health services, including hospitals, outpatient centers, primary care facilities, and public health institutions. Studies were selected based on the eligibility criteria defined in the research protocol [24] and summarized in Textbox 1. The recently updated version of the Consolidated Framework for Implementation Research (CFIR), a commonly used model to assess factors influencing implementation and to explain barriers and facilitators to implementation effectiveness [27,28], was used as a criterion for inclusion. Specifically, only studies that explicitly reported factors related to the CFIR domain of inner setting or process were considered eligible for inclusion (Textbox 2).

**Eligibility criteria.**

**Inclusion criteria**

Study design: Empirical studies illustrating the implementation of machine learning (ML)-based applications (eg, experimental/quasiexperimental, observational, hybrid, or simulation study designs, qualitative designs, case studies, etc)

Intervention: Analysis of the implementation of ML-based applications by at least covering factors related to the inner setting or process domain based on the Consolidated Framework on Implementation Research (CFIR)

Stakeholder groups: ML-based applications used at least by health care professionals (HCPs)

Setting: Hospitals, outpatients, and other community care settings

Timeframe: Studies published from 2013 until March 2023

**Exclusion criteria**

Study design: Effectiveness research study designs, literature reviews, commentaries, editorials, opinion articles, study protocols, studies collecting perceptions on implementation, and studies unrelated to specific ML-based applications

Intervention: Analysis of the implementation of logic- or knowledge-based applications (eg, expert systems) or ML-based applications with no considerations related to the inner setting or process domain

Stakeholder groups: ML-based applications targeting patients and other nonclinical stakeholders (eg, caregivers, policy makers, and regulators) only

Setting: All other settings, including home care

Timeframe: Studies published before 2013

**Domains of the Consolidated Framework for Implementation Research (CFIR).**

Innovation: Domain that collects the characteristics of the implemented object from a multi-faceted point of view.

Outer setting: Domain designed to capture factors that are inherent in the context where the organization exists.

Inner setting: Domain that encompasses the characteristics of the organization in which the innovation is implemented. It includes both structural attributes, which characterize the inner setting regardless of the implementation, and features, which are specific to the implementation.

Roles: Domain that refers to the individuals who have significantly contributed to the implementation and their characteristics.

Implementation process: Domain that collects all the information on the activities and strategies adopted to concretely implement the innovation.

### Information Sources

Literature searches were conducted in MEDLINE (PubMed), Scopus, and Web of Science and replicated in top-tier management journal databases. In addition, the reference lists of all included studies and of the reviews identified were scanned to ensure comprehensive coverage of relevant literature. Grey literature was not considered.

### Search Strategy

The search strategy was developed by the research team through an iterative process and was based on 4 main concepts: (1) AI; (2) implementation; (3) health care; and (4) study design. Multimedia Appendix 2 presents the search strings used for each database. The general term “artificial intelligence” was used broadly to encompass studies that address AI and ML as synonymous terms. The search was performed in April 2023.

### Study Selection and Data Collection Process

Two researchers (VA and LMP) screened the first 100 retrieved studies based on titles and abstracts. Once alignment over the inclusion and exclusion criteria was reached, the remaining records were independently screened by the 2 reviewers in equal parts based on the title and abstract. Disagreements over final inclusion were solved by a third researcher (FP). Studies deemed eligible for full-text reading were assessed in-depth (VA, LMP, and FP). Disagreements were resolved by dialogue with 2 additional researchers (GC and AC). The entire research team read all the studies included in the analysis. The data collection process was performed by 3 reviewers (VA, LMP, and FP) who extracted data using an ad hoc Microsoft Excel sheet preliminarily developed by the research team. To ensure consistency across reviewers, the extraction sheet was tested by each reviewer and recalibrated before starting the data collection process. Any disagreements were resolved by discussion with the research team, with final decisions reached by consensus.

### Data Items

Data items were extracted based on established classifications or schemes, when applicable [24]. These encompassed information on the paper (eg, journal of publication and publication year), ML application (eg, name, brief description, main practice of use, level of autonomy, and degree of integration with other technologies), and implementation process (eg, stage of implementation, geographical location, care setting, and specific unit of implementation). Furthermore, factors influencing the implementation process were assessed following the 5 domains of the updated version of the CFIR.

### Quality Assessment

Critical appraisal of the studies selected for data synthesis was performed using the Mixed Method Appraisal Tool (MMAT [29]), which has been designed specifically for systematic reviews that include heterogeneous studies, as it allows to assess the methodological quality of 5 types of study designs (ie, qualitative studies, randomized controlled trials, nonrandomized studies, quantitative descriptive studies, and mixed methods studies). Quality appraisal was performed by 2 researchers (VA and LMP), and disagreements were discussed and solved. The quality assessment represents a deviation from the protocol, which did not include this step.

### Data Synthesis

Given the significant heterogeneity across study designs, research objectives, and outcomes observed, as well as the expected predominance of qualitative studies, we opted for a thematic synthesis approach to capture and synthesize the salient attributes of the implementation process based on the CFIR constructs [30,31]. The analysis considered findings from the data extraction process as qualitative data and included summaries and interpretation of findings from the authors of the reviewed studies. Hence, direct quotes from participants were excluded in cases where the study employed qualitative data collection methods (eg, interviews).

We used both an inductive and a deductive approach. Following the 3 thematic synthesis steps, we initially reviewed each paper and highlighted relevant aspects through line-by-line coding to capture and collect key data. The coding process involved 3 reviewers (VA, LMP, and FP). To identify recurring topics, primary codes were then compared, organized, and labeled to derive descriptive themes reflecting their meaning. Descriptive themes were used to develop higher-level analytical themes. The formulation of descriptive themes and the following assignation to analytical themes were initially proposed by a researcher (LMP) and iteratively refined through discussions with 2 other researchers (VA and FP).

The higher-level analytical themes were subsequently deductively redefined by the entire research team within the constructs of the CFIR, which served as the final theoretical framework guiding our analysis.

## Results

### Study Selection

**Figure 1:**
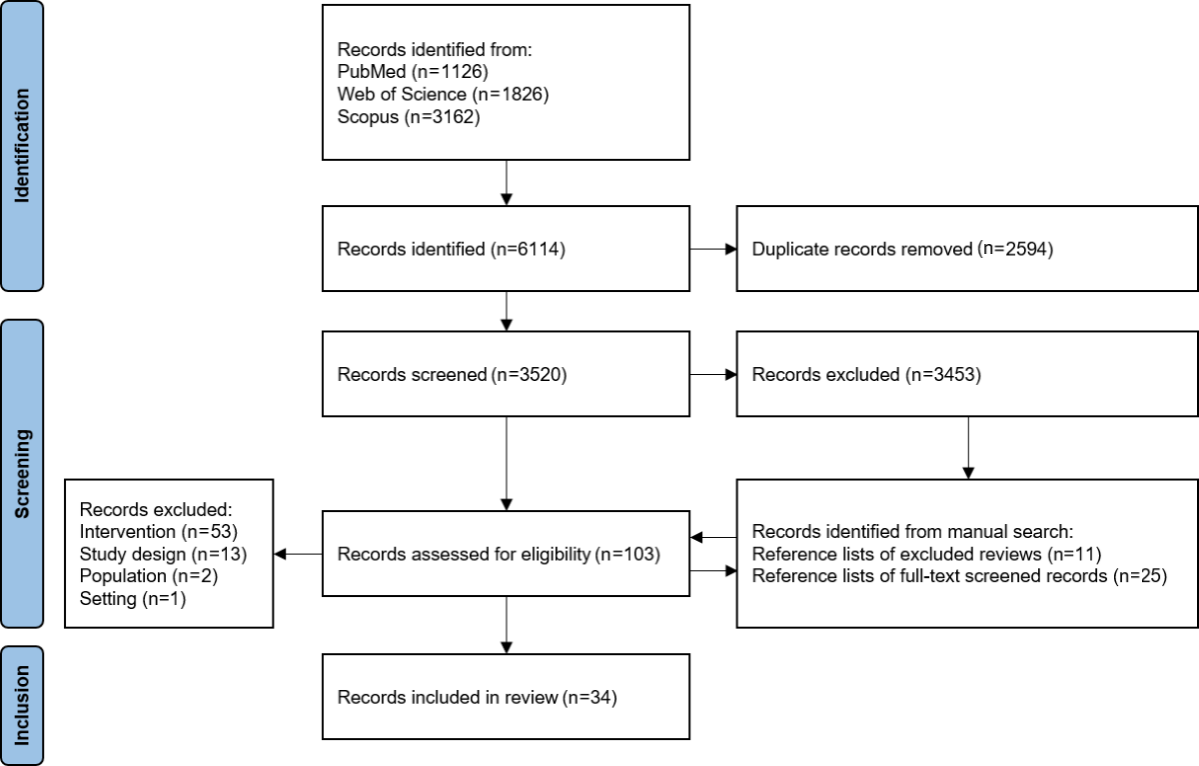
PRISMA (Preferred Reporting Items for Systematic Reviews and Meta-Analyses) flow diagram.

We retrieved 3520 unique records that were initially screened based on the titles and abstracts. A total of 67 records were deemed eligible for full-text screening (67/3520, 1.9%). Additionally, we identified 36 eligible records from a manual search of reference lists of excluded literature reviews and full-text screened records. Out of the 103 papers analyzed in full text, 69 were excluded and 34 were included in the review (34/103, 33.0%). The primary reason for exclusion was the focus of the intervention analyzed in the papers (53/69, 77%), as they either had a clinical or technical purpose without addressing factors related to implementation in an organizational setting or involved non-ML–based applications. Figure 1 provides an overview of the selection process and the reasons for exclusion.

### Study Characteristics

Most of the studies documenting the implementation of ML-based applications were set in the United States (18/34, 53%). Other locations included China (4/34, 12%); Canada, Brazil, and the Netherlands (each 2/34, 6%); and Italy, Spain, Norway, Korea, India, and Austria (each 1/34, 3%). Papers selected for data synthesis were relatively recent, with only 3 out of 34 (9%) published before 2019. Outlets were mostly clinical or in the field of information technology (IT) (30/34, 88%), while the remaining (4/34, 12%) focused on managerial or organizational studies. Most of the selected studies followed qualitative or mixed methods designs (22/34, 65%), often relying on methods such as interviews and case studies.

### Quality Assessment in Studies

Quality appraisal of the selected studies was performed using the MMAT. The studies were heterogeneous in terms of study design, and different MMAT questions were used to assess their quality. Overall, 18 studies leveraged the questions of qualitative studies, 7 of quantitative nonrandomized studies, 5 of quantitative descriptive studies, 3 of mixed methods studies, and 1 of quantitative randomized studies. Overall, the quality assessment suggested a medium-good quality of the studies, with only 12.5% of the assessment questions uncertain or unclear (“Can’t tell”). The detailed output of the quality appraisal is provided in Multimedia Appendix 3.

### Characteristics of ML Applications

Table 1 provides a general description of the ML-based applications implemented in the selected studies [32-65], while Multimedia Appendix 4 provides more detailed information on the characteristics of these applications. The most recurrent applications comprised predictive modeling algorithms, visualization tools, and alert-delivering mechanisms. All the applications identified by our search were clinical practice applications, according to the definitions from the European Parliamentary Research Service [66]. Moreover, none of the applications had decisional autonomy; therefore, all systems could be classified as clinical decision support systems (CDSSs).

**Table 1:** Overview of machine learning applications.

Authors	Year	Application name	Application output description	Implementation setting (unit)	Clinical workflow activity
Lee et al [32]	2015	—^a^	Prediction of patient characteristics, complaint types, and admission and readmission patterns	Hospital (ED^b^)	Prognosis
Hengstler et al [33]	2016	IBM Watson	Building hypotheses and evidence on cancer diagnosis	Hospital (oncology)	Diagnosis
McCoy & Das [34]	2017	InSight	Prediction of the risk of developing severe sepsis	Community hospital (ED, ICU^c^)	Prognosis
Bhattacharya et al [35]	2019	Niramai Thermalytix and iBreastExam (iBE)	Earlier detection of breast cancer	Hospital (radiology)	Diagnosis
Cruz et al [36]	2019	Savana	Recommendations for improving adherence to health care pathways	Primary care	Clinical/organizational workflow
Ginestra et al [37]	2019	EWS 2.0	Prediction of the risk of developing sepsis	Teaching hospital (non-ICU setting)	Prognosis
Gonçalves et al [38]	2020	Laura	Prediction of the risk of developing sepsis	Hospital (several units)	Prognosis
Sun & Medaglia [39]	2019	IBM Watson for Oncology	Decision-making support for personalized treatment planning	Hospital (oncology)	Treatment
Baxter et al [40]	2020	—	Prediction of unplanned readmission	Teaching and research hospital (unspecified)	Prognosis
Cho et al [41]	2020	DEWS (Deep-Learning-based Early Warning System)	Prediction of in-hospital cardiac events	Hospital (cardiology)	Prognosis
Frontoni et al [42]	2020	—	Production of indicators for quality-of-care processes of T2D^d^	Primary care	Clinical/organizational workflow
Hassan et al [43]	2020	Viz.ai	Detection of large vessel occlusions	Hospital (stroke unit)	Diagnosis
Romero-Brufau et al [44]	2020	—	Prediction of hospital readmission and formulation of targeted recommendations	Hospital (all units)	Prognosis and treatment
Sandhu et al [45]	2020	Sepsis Watch	Prediction of the risk of developing sepsis	Teaching hospital (ED)	Prognosis
Sendak et al [46]	2020	Sepsis Watch	Prediction of the risk of developing sepsis	Teaching hospital (ED)	Prognosis
Strohm et al [47]	2020	BoneXpert	Assessment of child maturation and bone age, and prediction of adult height	Hospital (radiology)	Diagnosis and prognosis
Xu et al [48]	2020	SensEcho	Classification of sleep stage, detection of sleep apnea, and recognition of abnormal ECG^e^ signals from a multi-sensor wearable device	Hospital (general and respiratory)	Diagnosis
Jauk et al [49]	2021	—	Prediction of the risk of developing delirium	Hospital (surgery, internal medicine)	Prognosis
Morales et al [50]	2021	Laura Digital ER	Detection of COVID-19 symptoms	Community	Diagnosis
Murphree et al [51]	2021	—	Treatment optimization and identification of likely-to-benefit patients for palliative care	Hospital (all inpatient units)	Treatment
Yao et al [52]	2021	3D CSAC-Net	Detection of mild COVID-19 pneumonia	Hospital (unspecified)	Diagnosis
Davis et al [53]	2022	Aidoc	Prediction of the risk of developing intracranial hemorrhage	Research hospital (radiology)	Prognosis
Henry et al [54]	2022	TREWS	Prediction of the risk of developing sepsis	Acute care hospital (inpatient acute units and ED)	Prognosis
Joshi et al [55]	2022	—	Prediction of the risk of developing sepsis	Community and teaching hospitals (several units)	Prognosis
Lebovitz et al [56]	2022	—	Image processing, segmentation, and classification for imaging diagnostics	Teaching hospital (radiology)	Diagnosis
Rushlow et al [57]	2022	—	Prediction of the risk of low left ventricular ejection fraction	Primary care	Prognosis
Schwartz et al [58]	2022	CONCERxN	Prediction of the risk of in-hospital deterioration	Teaching hospital (acute units and ICU)	Prognosis
Sibbald et al [59]	2022	Isabel	Differential diagnosis	Teaching hospital (ED)	Diagnosis
Singer et al [60]	2022	Low Bed Tool and Readmission Risk Tool	Prediction of reduced bed availability and prediction of the risk of readmission	Hospital (ICU, surgery, pediatrics)	Clinical/organizational workflow and prognosis
Wijnhoven [61]	2022	Sepsis Identification Speed	Prediction of the risk of developing sepsis	Teaching hospital (neonatology)	Prognosis
Zhai et al [62]	2022	Nu-CDSS	Formulation of recommendations for nurses’ diagnoses, interventions, and outcome evaluations	Teaching hospital (unspecified)	Clinical/organizational workflow
Pou-Prom et al [63]	2022	CHARTwatch	Early warning system designed to predict patient risk of clinical deterioration	Teaching hospital (general internal medicine)	Prognosis
Hinson et al [64]	2022	—	Estimation of the short-term risk for clinical deterioration in patients with or under investigation for COVID-19	Teaching hospital (ED)	Prognosis
Berge et al [65]	2023	Information System for Clinical Concept-based Search	Detection and classification of patient allergies	Hospital (anesthesia, ICU)	Diagnosis

^a^Not applicable.

^b^ED: emergency department.

^c^ICU: intensive care unit.

^d^T2D: type 2 diabetes.

^e^ECG: electrocardiogram.

In terms of settings, ML-based applications were mostly implemented within hospitals (29/34, 85%), including general, university, or teaching hospitals, academic medical centers, and research centers. A few studies (4/34, 12%) were based in a community or primary care setting. Within hospital settings, the most recurring implementation units were emergency departments (EDs) (11/34, 32%) and critical care units such as intensive care units (ICUs) (4/34, 12%), while in some studies, implementation occurred in multiple units or at the hospital level (5/34, 15%).

The clusters identified by Rajkomar et al [67] were used as a theoretical guide to classify the clinical workflow activities in which the ML-based applications were used. In 20 studies (59%), the ML tools supported prognosis. Many of these applications were designed to predict the risk of developing specific conditions such as sepsis (8/34, 24%), in-hospital deterioration (3/34, 9%), intracranial hemorrhage (1/34, 3%), or heart failure (1/34, 3%). Other applications predicted the risk of unplanned hospital admission or readmission (4/34, 12%). Ten papers (29%) illustrated applications for diagnosis, either as standalone computer vision tools to detect diseases from diagnostic imaging (eg, pneumonia from computed tomography [CT] scans, large vessel occlusions from CT angiograms, and child maturation from x-rays) or as diagnostic supports in emergency physician triage. Three papers (9%) illustrated applications for treatment optimization and personalization.

ML capabilities relate to clinical workflow activities, with forecasting (ie, the ability to find complex patterns in data and make predictions) being the most prevalent capability (19/34, 56%), as this function is typical of tools that predict the risk of an adverse event (12 of 34 forecasting tools were for prognosis). Computer vision was exclusively included in algorithms for diagnostic purposes, with all 6 computer vision tools intended for diagnosis.

As for the level of integration with existing technologies, 17 ML-based algorithms (50%) were embedded in electronic health records (EHRs) or similar platforms (ie, add-ons to the EHR software in use). Fourteen algorithms (41%) were standalone applications, fed either with internal or external data, including images or text. One application (3%) was embedded in hospital hardware technology, namely scanner machines [34]. Computer vision applications were always standalone applications provided as software to be installed within existing hardware (ie, hospital computers) and integrated with local picture archiving and communication systems (PACS).

The ownership of the algorithms was also assessed, revealing a division between applications purchased from commercial vendors (14/34, 41%) and those developed internally (12/34, 35%). The latter algorithms were often linked with the organizational setting, as 6 of these studies were carried out in teaching hospitals, academic medical centers, or research centers. Externally purchased applications were more common in other settings and exhibited greater diversity in terms of purposes, while homegrown tools were generally intended for prognostic purposes. In 8 studies, information on the name or the development process of the application was irretrievable, preventing the determination of algorithm ownership.

Details on the specific ML models employed were often missing, although it was possible to infer that 20 of the analyzed studies (59%) were based on supervised learning models such as random forest, decision tree, and logistic regression.

### Implementation Process Characteristics

**Figure 2:**
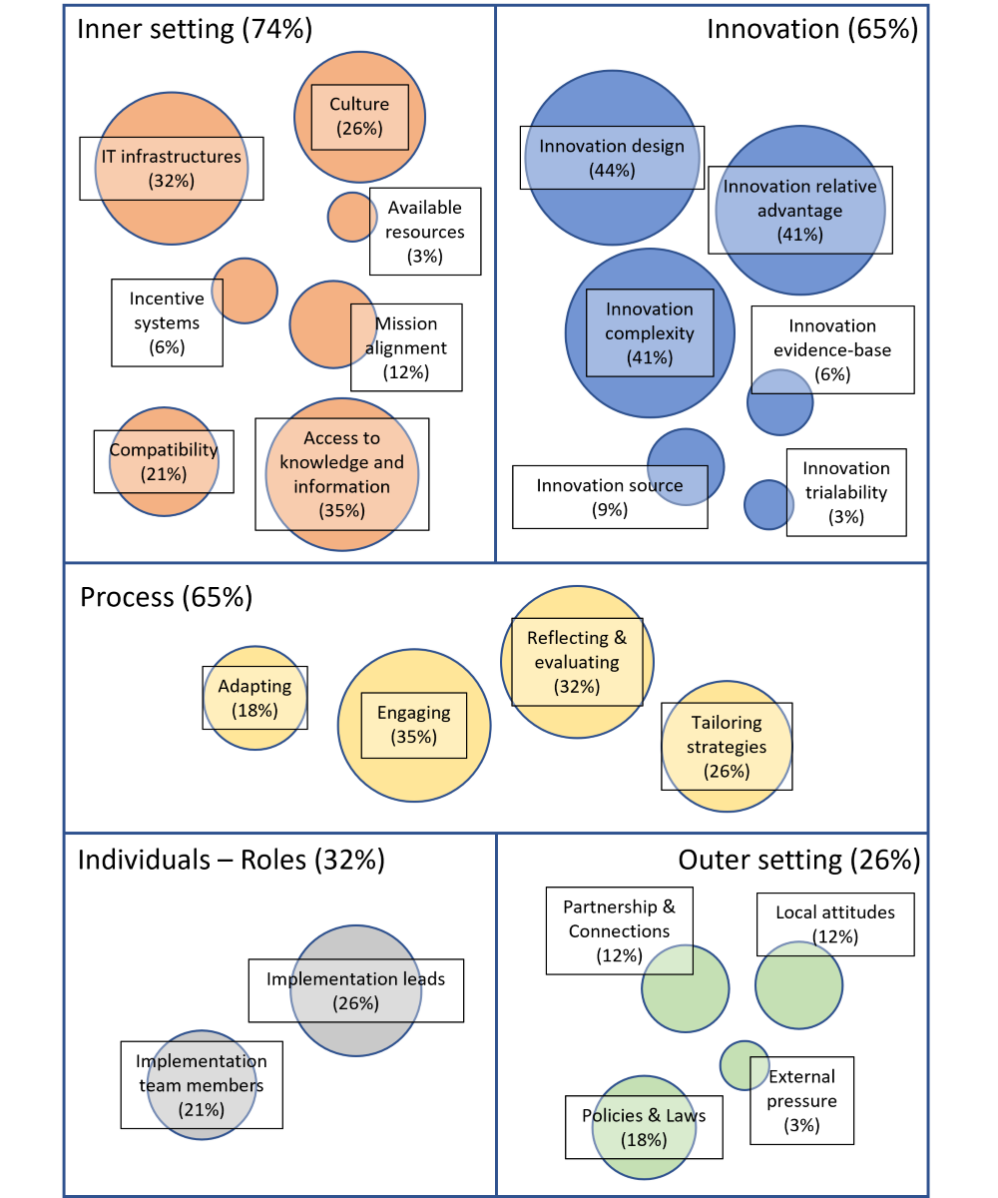
Relative importance of Consolidated Framework for Implementation Research (CFIR) constructs. The percentages represent the proportions of papers in which each construct and domain appears out of the 34 included in the review. The size of the bubbles corresponds to the frequency of occurrence of each construct.

This section presents the results of the thematic analysis, discussed following the 5 domains of the CFIR, namely innovation, outer setting, inner setting, roles, and implementation process. From the 34 studies analyzed, 222 quotes were extracted. Quotes were organized in 167 descriptive themes and 42 analytical themes. Analytical themes were finally embedded into 23 CFIR constructs. The detailed results of the coding process are presented in Multimedia Appendix 5. To provide a simplified overview of the coding process, Table 2 summarizes the analytical themes, their correspondence with CFIR constructs, and relative frequencies. The results are reported according to the frequency of information extracted on CFIR domains. The relative importance of CFIR constructs is presented in Figure 2.

**Table 2:** Analytical themes, constructs, and domains of the Consolidated Framework for Implementation Research (CFIR).

Construct		Analytical themes	Papers, n (%)	References
**Inner setting domain (n=25, 74%)**				
	A. Structural characteristics (A.2 IT infrastructure)	Integration with existing IT; Data governance; System infrastructure	11 (32)	[33,36,38,39,47,48,50,57,58,60,61]
	D. Culture	Professional habits; User perceptions	9 (26)	[32,37-40,45,47,59,62]
	F. Compatibility	Local workflow adaptation	7 (21)	[40,44,47,49,62,63,65]
	H. Incentive systems	Economic incentives; Organizational incentives	2 (6)	[42,56]
	I. Mission alignment	Organizational strategy; Organizational support	4 (12)	[39,47,55,62]
	J. Available resources	Resource reallocation	1 (3)	[32]
	K. Access to knowledge & information	Skills	12 (35)	[32,34,38-40,45,48,49,55,57,63,64]
**Innovation domain (n=22, 65%)**				
	A. Innovation source	Trust in the innovation source	3 (9)	[33,39,44]
	B. Innovation evidence base	Empirical evidence on added value	2 (6)	[47,49]
	C. Innovation relative advantage	Performance trust; Perceived cons; Perceived benefits	14 (41)	[37,39,40,43-45,47,55,56,58,59,61,62,65]
	E. Innovation trialability	Testing period	1 (3)	[63]
	F. Innovation complexity	Explainability	14 (41)	[32,33,37,39,44,45,49,54-56,58,59,61,65]
	G. Innovation design	Complementarity; Ease of use; Risks	15 (44)	[32,33,39,45,49,52-56,58,59,62,64,65]
**Process domain (n=22, 65%)**				
	E. Tailoring strategies	Framing; Tailoring	9 (26)	[33,38,45-47,54,55,57,62]
	F. Engaging	Early involvement of end-users; Professional buy-in; Iterative development	12 (35)	[33,38,45,46,51,55,57,58,60-63]
	H. Reflecting & evaluating	Feedback	11 (32)	[34,36,38,45,46,48,49,60-62,65]
	I. Adapting	Local data; Adaptability	6 (18)	[36,39,41,44,51,58]
**Individuals domain – Roles subdomain (n=11, 32%)**				
	E. Implementation leads	Implementation lead; Implementation team	9 (26)	[34,40,45-47,55,61-63]
	F. Implementation team members	Interdisciplinary teams	7 (21)	[45,46,51,55,61,62,64]
**Outer setting domain** **(n=9, 26%)**				
	B. Local attitudes	Patient acceptance; Public attitude	4 (12)	[33,35,39,50]
	D. Partnership & connections	Interinstitutional partnerships; Public-private partnerships	4 (12)	[42,47,50,61]
	E. Policies & laws	Medicolegal issues; Medical device regulation; Guidelines; Data protection	6 (18)	[33,39,46,47,50,61]
	G. External pressure	Peer influence	1 (3)	[54]

### Inner Setting

The inner setting domain was the most frequently described, with 25 studies mentioning at least one construct from this domain as relevant to explaining the implementation process of the ML application. The most recurrent constructs were access to knowledge and information (12/34, 35%), IT infrastructure (11/34, 32%), and culture (9/34, 26%).

First, the *access to knowledge* construct aligned with the topic of skills. Studies emphasized the importance of providing end users with access to training programs on both hard and soft skills before implementation [48,49,55,64], including computer and technical literacy linked with the complexity of the application’s functioning [57,63], and the medical domain that the application addresses [45]. The latter referred to dimensions, such as communication, empathy, and ability to listen, especially when different HCPs were involved in the implementation process [38,39,45].

Second, the *IT infrastructure* construct encompassed 2 prominent themes. The first broadly concerned data management and data governance. Themes, such as data collection and quality [39,58,59], security [33], availability [36,38], and sharing [39,61], were highly described as challenges for the adoption of the application. There were also significant references to building IT infrastructure [61] and to the need to integrate new technologies with existing IT systems (eg, EHRs). While integration promoted ease of use by reducing the need for manual inputs [47,48,50], some argued for the ML application not to directly populate EHRs in order to preserve HCP autonomy and prevent medicolegal accountability [59].

Finally, the construct of *culture* was articulated into the themes of professional habits and alignment of perceptions among stakeholders. The impact of introducing ML applications on professional habits was significant as this affected how professionals work, interact, and make decisions [62]. For instance, the habit of working without technological support was considered a barrier to implementation [39,40]. The need for gradual changes in professional habits was seen as a factor that could hinder the adoption of ML applications in settings with high job rotations (eg, teaching hospitals) [32]. Additionally, ML applications often are not tailored to local workflows and do not consider the different approaches of professionals in diverse contexts [39]. The other theme related to culture was that of perceptions and expectations among different internal stakeholders (eg, management, physicians, nurses, and technical staff). Misalignments among these stakeholders were common, particularly regarding trust in ML in general [45,47] or the expected target users (eg, residents vs expert physicians) [45,59].

### Innovation

Innovation and its characteristics were among the most frequently mentioned domains (22/34, 65%), with 3 constructs absorbing a significant portion of relevant descriptive themes: innovation design (15/34, 44%), relative advantage (14/34, 41%), and complexity (14/34, 41%).

First, *innovation design* encompassed themes related to the applications’ design and functioning, including the types of human-machine interactions, as well as the associated risks. The most recurrent themes within this construct revolved around ease of use and intuitive design [33,45,49,62]. The former was often linked to minimizing manual interventions, such as data input [49,52], and was also associated with dimensions of trust in the applications, such as trust in the process and the cognitive burden for HCPs, in the form of fatigue from overalerting [33,62], which could be a barrier to professional buy-in [55]. Some studies explicitly cited the theme of human-centered design as a development framework that starts with the assessment of end users’ needs and the environment in which the ML application will be used [54,64,68]. Another recurrent theme was the human-machine complementarity. For HCPs, it was often important to maintain a sense of control over the application and not perceive it as an attempt at uncontrolled substitution and automation [32,33,56,65]. Human-machine complementarity was also associated with fewer disruptions to established workflows, enhancing the overall benefits associated with the use of ML applications [45,53]. Moreover, complementarity could increase trust in the application from both a micro-perspective (eg, its functioning) [33,54] and macro-perspective (eg, the purpose of the application and the reasons for choosing to integrate ML within a clinical context) [56,58,65]. The risks of ML use in decision-making processes also emerged. These included the risk of automation, in terms of overreliance on ML recommendations [39], and the risk of bias, tied to the underlying data and training model of the ML application [59]. Moreover, the potential negative consequences of automation risk on clinical ability were mentioned [54].

Second, the *relative advantage* revolved around the perception of benefits and costs associated with the use of ML, as well as factors influencing trust in its performance. The most frequently perceived benefits were related to the organizational dimension, in terms of optimization of the workflow resulting from the elimination of unnecessary steps [43], increased attention from end users to all cases managed by the application [45], and enhanced interactions among physicians and other HCPs [62]. Conversely, references to the economic impact were ambiguous. On the one hand, faster decision-making could be considered a potential advantage [47], and on the other hand, human-machine interaction could lead to a loss of efficiency compared to human intervention only [40,56]. Another barrier to professional buy-in is that the perceived poor ability of the application to take contextual factors into account calls into question its clinical relevance. Among the perceived advantages, trust in the application’s performance and its determinants were often commented on. For the analysis, we adopted the concept of trust as defined by Hengstler et al [33] who distinguished between trust in technology and trust in those who produce it (ie, the source of innovation). This definition further divided trust in technology into 3 dimensions: trust in performance, focusing on the accuracy and consistency of the output; trust in the process, concerning the understanding of the reasoning behind a given output; and trust in the purpose of the innovation to be implemented [16,44]. Concordance significantly influences trust in performance, with a greater difference between human judgment and machine recommendation associated with a lower level of trust in the recommendation [44,47,58]. Similarly, recommendations that did not arrive in a timely or adequate manner negatively influenced trust in performance [37,44]. Additionally, trust in performance could be fueled by experience, the application’s ability to identify cases missed by humans, and the consistency over time of recommendations [47].

Third, *innovation complexity* highlighted the concepts of explainability and opacity as distinctive features of ML models. Many studies were consistent in identifying algorithm complexity as the primary barrier to trust in the process underlying the generation of an ML output. This is even more true when nonmedical professionals (eg, nurses) interact directly with the ML application [45]. Facilitating interpretability, explainability, or cognitive compatibility was mentioned as a way to promote transparency, trust among HCPs, and professional buy-in [49,55,56,58,59,65].

### Implementation Process

The reviewed articles often mentioned the characteristics of the implementation process (22/34, 65%), with a particular emphasis on the constructs of stakeholders engaging (12/34, 35%), reflecting and evaluating (11/34, 32%), and tailoring strategies (9/34, 26%).

Attracting and encouraging the *participation of different stakeholders* in the implementation process emerged as a recurring theme. The practice of early involvement of end users was frequently cited not only during the implementation process but also throughout the development phase [33,38,45,46,58,61,63]. This was positively associated with trust in the innovation’s purpose [46], the application’s functioning [58,61,62], and the ease of use of its design [45]. During the implementation phase, stakeholder engagement was linked to evident benefits, such as improvement in the implementation climate [46], greater willingness to adopt the role of implementation leader [38], greater professional buy-in [51,55], and better iterative collection of information and feedback [60]. Conversely, the absence of engagement was seen as a barrier to successful implementation, potentially leading to increased resistance toward the innovation among end users [62].

In the construct of *reflecting and evaluating*, feedback and feedback loops emerged as recurring topics, with many studies underscoring the importance for both ML developers and implementation teams to incorporate end users’ feedback on either technical issues, system design, or clinical needs [34,36,38,49,62,65]. Some studies noted that feedback collection extended beyond implementation, with structured feedback loop processes integrated into routine use [46,48]. Regardless, feedback collection was described as an iterative activity [46,48,60,61], which also positively influenced professional buy-in [47]. However, a critical point raised was that end-users may lack the necessary technical skills to provide feedback conducive to improvement [62].

Two additional recurring constructs were tailoring strategies and adapting. The former referred to actions addressing barriers and leveraging facilitators, while the latter involved modifying the innovation itself to best fit the context in which it was inserted.

Among *tailoring strategies*, the importance of effectively communicating the implementation efforts was often highlighted. Some works referred to the need for clearly framing communication around the expected benefits, positively affecting trust in ML-based innovations [33,38,45-47,54] and trust in the innovation source [33], and fostering greater professional buy-in [55]. Another aspect of framing was related to the terminology used, asserting that using terms supporting concepts, such as “assistant” and “support” had a favorable impact on end users’ trust toward ML-based innovations [46,47] and the innovation source [33].

In terms of *adapting*, the first theme involved the need for collected feedback to be effectively incorporated into the application, adapting systems to the local context of implementation [36,58]. The second involved the issue of data, emphasizing the importance that the model is effectively trained and adapted to the cases treated in the clinical context in which the application will be used before deployment. The absence of this aspect was perceived as a barrier to trust in the ML application’s performance [39,41,44,51].

### Individuals: Roles

The subdomain of roles was less frequently observed (11/34, 32%) and encompassed 2 constructs: implementation leads (9/34, 26%) and implementation teams (7/34, 21%).

The former referred to the individual or group that guided and oversaw the implementation process, and their presence was generally considered a positive factor for implementation as it contributed to establishing a favorable implementation climate [47]. Individual implementation leaders were often referred to as champions. Although it may theoretically involve figures that emerge from bottom-up processes, all works referring to this role mentioned a top-down identification [45,47,62,63]. Implementation teams were observed as well in the form of quality improvement teams [34], AI governance committees [40,46,63], or interdisciplinary teams of HCPs, software engineers, developers, IT specialists, and other figures [46,51,55,64].

### Outer Setting

The outer setting domain emerged poorly in the reviewed studies (9/34, 26%), particularly in the form of 3 constructs: policy and laws (6/34, 18%), local attitudes (4/34, 12%), and partnership and connections (4/34, 12%).

In the *policy and laws* construct, 3 main themes emerged. The first concerned the medicolegal responsibility for decisions made using an ML application [39,61]. The second pertained to regulatory and certification aspects, with recognition of the application as a medical device seen both as a factor positively influencing trust in the application [33] and as a barrier to utilization [46]. Regulations on personal data protection were also considered implementation challenges [47]. Regarding policies, the only theme mentioned was the relevance of national policies and guidelines to create a common framework for the implementation of ML applications [50].

*Local attitudes* were societal expectations and beliefs on the use of ML applications. Cultural aspects, innovation attitudes, and public expectations could influence the acceptability of ML [35,39,50]. Equally relevant for acceptance was the visibility of the application (ie, how noticeable and observable an innovation is to the public), which influences how organizations foster innovation trust [33,69].

Within the *partnership and connections* construct, building partnerships with scientific societies and professional communities was considered a facilitator for implementation, as these can act as knowledge platforms or hubs [42,47]. Professional communities and peers could also trigger external pressure that may positively impact the willingness to implement ML applications [54]. Establishing development networks across hospitals and health care facilities was a relevant factor for the increased reliability of the application, providing the opportunity to leverage larger datasets, which are known to end users [61]. Moreover, forging public-private partnerships was deemed a useful step for implementation, to leverage expertise not always available within public health care organizations [50,61].

## Discussion

### Review of the Main Findings

This work aimed at synthetizing extant academic knowledge on the implementation of ML-based applications in clinical practice, focusing specifically on the characteristics of the innovation and on the processes and strategies employed by health care organizations to ensure their successful implementation.

We identified 34 studies reporting on the implementation process of ML applications, all of which were CDSSs frequently based on supervised learning models in the form of predictive algorithms, visualizations, and alert-delivering tools. Overall, half of the observed applications were integrated into hospital information systems as add-ons to the EHR infrastructure. ML-based applications were mainly implemented in hospital settings and supported prognostic activities, although a relevant portion was intended for diagnosis. Among the diagnostic applications, those based on computer vision were either standalone software or embedded in the hospital hardware technology. Algorithms could be clustered into 2 groups: those internally developed, prevalently by university hospitals and academic medical centers and typically with a prognostic purpose, and those purchased from commercial vendors, which are more heterogeneous in terms of purposes and functions.

Furthermore, our analysis enabled us to scrutinize the characteristics of the implementation processes of ML-based applications, gathering pertinent insights relevant to their successful integration within health care organizations. Through the theoretical lens of the CFIR, we identified a predominant emphasis on 3 key domains: inner setting, innovation characteristics, and process dimension. First, evidence from the inner setting domain highlighted the importance of addressing IT infrastructure and data management challenges, as well as the necessity of fostering an organizational culture that favors the implementation of ML-based applications. Second, in terms of innovation design, the concept of human-machine complementarity was recurrent, highlighting the importance of integrating ML-based applications into existing workflows to enhance overall benefits and foster trust by ensuring HCPs maintain a sense of overall control. In the process domain, studies emphasized the importance of fostering early stakeholder engagement during the development and preimplementation phases, adapting strategies to local contexts, and initiating reflection and evaluation activities to support continuous improvement based on feedback loops. Conversely, while the complexity inherent in ML models in terms of algorithm opacity was largely acknowledged, we found limited investigation into effective mitigation strategies to tackle these challenges.

### Comparison With Prior Work

Different from prior work encompassing logic-based and rule-based applications [10,70,71], our study focused exclusively on ML-based applications. While the frequency and relative significance of various application types are not directly comparable with those observed in the cited works, other recent reviews have adopted a similar approach to ours. In their scoping review, Chomutare et al [21] identified 19 studies on the implementation of AI applications powered by ML, highlighting a variety of solutions across medical fields and tasks within the clinical workflow. Similarly, Tricco et al [22] explored how implementation science strategies can facilitate the implementation of ML tools, but their work also included studies with effective research designs, thereby adopting a partially different approach from that of this work. Our review expanded the number of included studies, confirming the multitude of diverse applications of ML in clinical practice. The only condition for which we observed a conspicuous number of studies was sepsis, a dysfunction accounting for around 20% of deaths worldwide [72], for which ML-based applications are proliferating [73], although no definitive causal link with reduced mortality has been demonstrated to date [74]. Our search identified 8 studies on sepsis, showcasing the potential attributed to ML-based applications in supporting the timely identification of hospital-acquired conditions. On a similar note, a recent review encompassing over 10,000 ML applications in health care settings corroborated the relevance of prognostic algorithms among those in use [75].

Consistent with previous research [21,70], most of the included papers presented cases of real-world implementation rather than proper implementation studies on the later phases of rollout, often covering only a few aspects of the implementation process.

While we hypothesized that distinct implementation strategies would be prevalent based on the characteristics of ML-based applications, we only observed limited distinctions based on the types of clinical applications (prognostic, diagnostic, or therapeutic purposes) or their development process (internal development vis-à-vis external acquisition and adaptation).

For instance, the integration with existing IT infrastructure introduced ambiguity in the context of diagnostic applications, where such integration may be perceived as a risk with medicolegal implications [50,59]. On the other hand, for applications with nondiagnostic purposes, integration with existing IT systems was viewed as a positive factor for ease of use [47,62].

Other elements appeared relatively more pronounced in applications provided by external providers. This included perceived risks associated with application design (eg, overreliance, automation, and bias) [39,54,59], considerations regarding complementarity with HCPs [33,53,54,56], and aspects related to explainability. As such, exploring whether and how different application types entail different implications for their effective integration into clinical practice might be a valuable suggestion for future research.

Just like the report by Chomutare et al [21], our work confirmed that the outer setting domain was largely overlooked, although prior studies have highlighted the importance of external factors, such as data privacy and security laws, ethical issues, regulatory frameworks, and medical liability, in implementing ML applications in clinical practice [5,76-78]. The limited relevance of such a domain in our sample may stem from 2 reasons. First, due to the nature of the included studies, only a few frameworks that were used accounted for elements beyond the organizational setting in which the implementation occurred. Factors associated with the outer setting may be more frequently highlighted in implementation processes perceived as unsuccessful, which are less often reported in the scientific literature. Second, since the primary studies predominantly involved HCPs, they did not incorporate managerial and policymaker perspectives. In fact, when the outer domain perspective was explored, nonclinical stakeholders were often involved [39,47,61]. Furthermore, Hogg et al [10] suggested prioritizing the perspective of non-HCP stakeholders in primary studies to enhance the understanding of implementation processes at a broader level, which may serve as a further valuable suggestion for future studies.

### Implications for the Implementation of ML-Based Applications: A Focus on Trust

The importance of trust, particularly within the physician-patient relationship, has been heightened by the advent of digital health, especially with innovations, such as ML applications that heavily rely on data [79]. ML applications based on computational models are often characterized as opaque (ie, black boxes), introducing an extra layer of complexity to the trust relationship between end users and technological innovations [80]. A recent review by Adjekum et al [79] categorized factors influencing trust in digital health systems into personal, technological, and institutional elements. Building upon the concept of trust as articulated by Hengstler et al [33], our work contributes to understanding the determinants of trust in facilitating the implementation of ML-based applications in health care organizations.

We observed that the characteristics of the innovation itself significantly challenged trust in the performance of ML-based applications. The complexity and opacity of the underlying models constitute primary barriers to trust, with trust in performance further influenced by system design elements such as ease of use, the nature of the HCP-machine interaction, and the timeliness and consistency over time of recommendations. Additionally, considerations regarding data governance for internally developed applications and the reputation of the technology provider for procured solutions further influence trust in the performance of these applications. However, as trust primarily remains a human-led process, factors beyond mere technical and mechanical characteristics influence trust in ML.

While most of the observed implementation strategies were essentially ML agnostic, addressing the issue of clinician trust should theoretically require dedicated ML-specific processes. Our review highlights potential ways to enhance the application-perceived reliability of ML applications. On the one hand, tailoring and adaptation strategies, early end-user engagement, and appropriate framing of ML-based applications as decision-support tools might favor HCP trust in both the application’s performance and its purpose [21]. On the other hand, specific tailoring strategies should be adopted to increase the explainability of nontotally interpretable models [13]. For instance, Jauk et al [49] enhanced clinical reasoning using a web application presenting relevant features from ML modeling, Davis et al [53] allowed radiologists to interact with the ML system by showing the types and locations of the abnormalities identified by the algorithm, and Henry et al [54] decided to delay alerts until the first verifiable symptoms were present in an attempt to increase acceptance.

However, these tailoring strategies may not be practicable when ML systems reach opacity levels that render the interpretation of their outputs impracticable. In such cases, other contributions have emphasized the need to highlight the level of actionability of ML models, in terms of their ability to enhance medical decision-making compared to clinical judgment alone, to power trust [81].

An additional contribution to enhancing trust may be achieved through continuous HCP involvement. This involvement, which generates engagement and professional buy-in, is equally significant for the successful implementation of these innovations. In the realm of digital health interventions, while there is frequent emphasis on patient engagement in the design of solutions, the empowerment of HCPs is often overlooked [82,83]. Active involvement of HCPs and frequent communications to raise awareness have been unambiguously identified among the most common enablers of trust in previous reviews on the implementation of ML applications [21,22,74,84]. This may facilitate the implementation of innovations by improving the implementation climate for reducing resistance to change and mitigating specific barriers associated with the complexity of ML models and the reliability of the recommendations they produce.

### Limitations

This study has some limitations that should be considered when interpreting our findings. First, the rapidly evolving nature of the field of ML and the exponential growth of newly published studies posed challenges in managing the vast volume of retrieved records. To address this, our search strategy incorporated a supplementary block of keywords focused on “study designs,” which may have excluded certain relevant articles. Additionally, our emphasis on peer-reviewed studies introduced a potential bias, as ML-based applications reported in the scientific literature may only represent a subset of implemented systems. This could impact the generalizability of our findings, as acknowledged in similar studies such as the study by Sharma et al [70]. Lastly, the decision to include only papers published in English might have led to the exclusion of valuable sources published in other languages, limiting the comprehensiveness of our review.

### Conclusions

Despite a relative dearth of primary studies on the implementation of ML applications in health care organizations, the available evidence reveals the abundance and heterogeneity of factors involved when ML applications are introduced in routine clinical practice. While certain elements, such as complexity and trust, tend to emerge as distinctive factors for ML applications, many other aspects reflect what is already known about the implementation of digital technologies, particularly traditional CDSSs.

Further research is needed to bridge the gap between the theoretical potential of ML and its actual use in health care organizations. Identifying the distinctive factors that can facilitate its implementation will build theoretical and practical knowledge for health care practitioners, ultimately promoting the uptake of ML in routine clinical practice.

## Data Availability

Data collected as part of this review will be made available by the corresponding author upon reasonable request.

## Appendix

Multimedia Appendix 1 PRISMA (Preferred Reporting Items for Systematic Reviews and Meta-Analyses) checklist.

Multimedia Appendix 2 Search strategy.

Multimedia Appendix 3 Quality assessment.

Multimedia Appendix 4 Characteristics of the studies and applications.

Multimedia Appendix 5 Coding process.

## Abbreviations

AI: artificial intelligence
CDSS: clinical decision support system
CFIR: Consolidated Framework for Implementation Research
CT: computed tomography
ED: emergency department
EHR: electronic health record
HCP: health care professional
ICU: intensive care unit
IT: information technology
ML: machine learning
MMAT: Mixed Method Appraisal Tool
