# Supplementary material for: Implementation of Machine Learning Applications in Health Care Organizations: Systematic Review of Empirical Studies
Source: J Med Internet Res. 2024 Nov 25;26:e55897. doi: 10.2196/55897 (PMC11629039; doi:10.2196/55897)
Supplement: Multimedia Appendix 2 [file jmir_v26i1e55897_app2.docx]

**SEARCH STRATEGY**

**SCOPUS**

( ( ( ( TITLE-ABS-KEY ( "artificial intelligence" OR "machine learning" OR "deep learning" OR "natural language processing" ) AND TITLE-ABS-KEY ( "implementation" OR "adoption" OR "diffusion" OR "uptake" ) AND TITLE-ABS-KEY ( "health*" OR "therap*" OR "patient*" OR "clinic*" OR "medic*" OR "doctor*" OR "hospital*" ) AND TITLE-ABS-KEY ( "empirical" OR "trial" OR "quantitative" OR "qualitative" OR "interview*" OR "case stud*" OR "case report*" OR "field stud*" OR "field research*" OR "mixed-method*" OR "mixed method*" OR "focus group*" OR "observational stud*" OR "hybrid design" OR "hybrid stud*") ) AND PUBYEAR > 2013 ) ) )

**PubMed**

(("artificial intelligence"[Title/Abstract] OR "machine learning"[Title/Abstract] OR "deep learning"[Title/Abstract] OR "natural language processing"[Title/Abstract]) AND ("Implementation"[Title/Abstract] OR "Adoption"[Title/Abstract] OR "Diffusion"[Title/Abstract] OR "Uptake"[Title/Abstract]) AND ("health*"[Title/Abstract] OR "therap*"[Title/Abstract] OR "patient*"[Title/Abstract] OR "clinic*"[Title/Abstract] OR "Care"[Title/Abstract] OR "medic*"[Title/Abstract] OR "Doctor"[Title/Abstract] OR "Hospital"[Title/Abstract]) AND ("empirical"[Title/Abstract] OR "quantitative"[Title/Abstract] OR "qualitative"[Title/Abstract] OR "trial"[Title/Abstract] OR "interview*"[Title/Abstract] OR "case stud*"[Title/Abstract] OR "case report"[Title/Abstract] OR "field stud*"[Title/Abstract] OR "field research"[Title/Abstract] OR "mixed method*"[Title/Abstract] OR "mixed method*"[Title/Abstract] OR "focus group*"[Title/Abstract] OR "observational stud*"[Title/Abstract] OR "hybrid stud*"[Title/Abstract] OR "hybrid design"[Title/Abstract])) AND (2014:2023[pdat])

**Web of Science**

"Artificial Intelligence" OR "Machine Learning" OR "Deep Learning" OR "Natural Language Processing" (Topic) AND Implementation OR Adoption OR Diffusion OR Uptake (Topic) AND Health* OR Therap* OR Patient* OR Clinic* OR Care OR Medic* OR Doctor OR Hospital (Topic) AND empirical OR quantitative OR qualitative OR trial OR interview* OR "case stud*" OR "case report" OR "field stud*" OR "field research" OR mixed-method* OR "mixed method*" OR "focus group*" OR "observational stud*" OR "hybrid stud*" OR "hybrid design" (Topic) and 2023 or 2022 or 2021 or 2020 or 2019 or 2018 or 2017 or 2016 or 2015 or 2014 (Publication Years)
